# Whole-Genome Sequencing Reveals Multiple Subpopulations of Dominant and Persistent Lineage I Isolates of *Listeria monocytogenes* in Two Meat Processing Facilities during 2011–2015

**DOI:** 10.3390/microorganisms10051070

**Published:** 2022-05-23

**Authors:** Elton Burnett, Zuzana Kucerova, Molly Freeman, Sophia Kathariou, Jessica Chen, Monica Smikle

**Affiliations:** 1 Institute of Parasitology, McGill University, 2111 Lakeshore Road, Montreal, QC H9X 3V9, Canada; 2 Department of Microbiology, University of the West Indies, Mona, Kingston 7, Jamaica; smiklemonica@gmail.com; 3 Centers for Disease Control and Prevention (CDC), 1600 Clifton Road, Atlanta, GA 30329, USA; zik0@cdc.gov (Z.K.); evy7@cdc.gov (M.F.); lly3@cdc.gov (J.C.); 4 Department of Food, Bioprocessing and Nutrition Sciences, North Carolina State University, Raleigh, NC 27695, USA; skathar@ncsu.edu

**Keywords:** *Listeria monocytogenes*, lineage I, MLST, clonal complex, PFGE

## Abstract

*Listeria monocytogenes* is a foodborne pathogen with a highly clonal population structure comprising multiple phylogenetic sub-groups that can persist within food processing environments and contaminate food. The epidemiology of *L. monocytogenes* is well-described in some developed countries; however, little is known about the prevalence and population structure of this pathogen in food and food processing environments located in less developed regions. The aim of this study was to determine the genetic characteristics and clonal relatedness of *L. monocytogenes* that were isolated from two Jamaican meat processing facilities. Of the 37 isolates collected between 2011 and 2015, only a single lineage II isolate was recovered (serotype 1/2c), and the remaining were lineage I isolates representing serotypes 4b, 1/2b, 3b, and two untypeable isolates. Pulsed-field gel electrophoresis (PFGE) delineated isolates into seven pulsotypes, and whole-genome sequencing (WGS) categorized most isolates within one of three clonal complexes (CC): CC2 (N = 12), CC5 (N = 11), and CC288 (N = 11). Isolates representing CC1 (N = 2) and CC9 (N = 1) were also recovered. Virulence-associated genes such as *inlA* and the *LIPI-3* cluster were detected in multiple isolates, along with the stress survival islet cluster-1 (SSI-1), and benzalkonium (*bcrABC*) and cadmium (*cad1, cad2, cad4*) resistance cassettes. Multiple isolates that belong to the same CC and matching PFGE patterns were isolated from food and the environment from both facilities across multiple years, suggesting the presence of persistent strains of *L. monocytogenes*, and/or constant re-entry of the pathogens into the facilities from common sources. These findings highlight the ability of lineage I isolates of *L. monocytogenes* to colonize, persist, and predominate within two meat-producing environments, and underscores the need for robust surveillance strategies to monitor and mitigate against these important foodborne pathogens.

## 1. Introduction

*Listeria monocytogenes* is an intracellular pathogen that causes sporadic foodborne infections and outbreaks in humans [1,2]. Invasive forms of the disease can result in fetal abortion and CNS complications, accompanied by a mortality rate above 20% in susceptible populations [3,4]. Approximately 1600 cases of human infections are reported annually in the United States of America [5], costing over USD 2 billion [6,7,8]. In addition, reported *L. monocytogenes*-related infections averaged 1563 annually in European Union member states during 2007–2012 [9]. *L. monocytogenes* comprises 13 serotypes and 4 lineages (I, II, III, and IV) [7]. Serotypes 1/2a (lineage II), and the lineage I serotypes 1/2b and 4b, cause >90% of clinical infections [2,10]. Serotypes 4b belongs to lineage I and is isolated from human infections with the highest frequency. However, a gradual increase in the isolation rate of serotype 1/2a from human cases has been observed in Canada and Europe [11,12,13,14,15]. Furthermore, serotype 4b isolates are also responsible for causing a majority of the global sporadic and outbreak incidents, indicating a comparatively increased level in pathogenicity [1,15]. Interestingly, lineage II isolates are most frequently recovered from food and food processing environments, despite showing a lower prevalence in human disease [1,16,17].

*L. monocytogenes* can colonize and persist in food processing facilities, contaminating food and equipment and posing a health risk to end consumers [18,19,20]. The survival and persistence of *L. monocytogenes* in unfavorable environmental conditions is mediated by the expression of multiple resistance- and stress-associated factors. For example, the stress survival islet-1 (SSI-1) increases tolerance to oxidative stress, bile, and low pH [21,22,23]. Sanitizers that contain quaternary ammonium compounds (QAC) are widely used to help maintain good sanitation in food processing environments; however, QAC resistance across several bacterial species is well documented [24]. Furthermore, QAC resistance is largely mediated by the *bcrABC* resistance cassette, which is located on plasmid pLM80 in some persistent isolates [25,26]. Heavy metal resistance also appears to promote environmental persistence in *L*. *monocytogenes*, and is conferred by the *cadAC* efflux cassette comprising *cadA1-4* [27,28,29,30].

Food is the major vehicle for *L. monocytogenes* infections [5] and ready-to-eat (RTE) meat, fresh fruits, and dairy products are frequently linked to outbreaks and sporadic disease [31,32,33]. Many North American and European countries have monitoring and surveillance programs for *L. monocytogenes* in food production and storage [34,35], as well as tolerance limit guidelines for various food types. This facilitates robust risk analysis and intervention strategies during outbreaks. Additionally, a zero-tolerance policy is enforced against *L. monocytogenes* contamination of RTE foods in the United States (Shank, F.R., 1996), while a maximum of 100 colony forming units (CFU) is allowed per 25 g of RTE meat, within the specified shelf-life, in European Union countries (Koutsoumanis, K., 2007). Limited resources often restrict surveillance of the pathogen in less developed countries, leading to its under-reporting [36]. For example, foodborne pathogens are actively monitored in many Caribbean countries, but reporting and diagnostic deficiencies still exist [37,38]. Overall, reports on the prevalence of *L. monocytogenes*-related incidents have been few from this region. However, data from Trinidad showed that *L. monocytogenes* and other *Listeria* spp. were isolated from RTE, raw meat, food animals, and food processing environments, with at least one documented recall linked to contaminated RTE meat [39,40,41,42]. A recent outbreak involving contaminated ice cream made in the USA also prompted a recall that was extended to Caribbean countries [43]. This further highlights potential routes of *L. monocytogenes* transmission between trading partners, and the need for robust monitoring and surveillance efforts in the Caribbean and neighboring territories.

The global population structure and epidemiology of *L. monocytogenes* is well-described; however, only limited information is available from the Caribbean region. This study uses a molecular approach including pulsed-field gel electrophoresis (PFGE) and whole-genome sequencing (WGS) to characterize *L. monocytogenes* isolates from two Jamaican meat processing facilities.

## 2. Materials and Methods

### 2.1. Sample Collection

The samples included in this study were randomly collected from two meat processing facilities (A and B), both located in Kingston, Jamaica. Access to the facilities was inconsistent and allowed only at the operators’ discretion. Facility A was sampled between November 2011 and December 2012, and facility B was sampled between August 2011 and July 2015. Both facilities process uncooked fish, pork, poultry, and beef. Environmental samples included swabs of food contact surfaces (knives, conveyor belts, mixers, and work tables) and non-food contact surfaces (floors, drains, door handles, and drain pans).

Meat samples were screened for *L. monocytogenes* as outlined in the Bacterial Analysis Methods manual (www.fda.gov/Food/FoodScienceResearch/LaboratoryMethods/ucm071400.htm, accessed on 1 January 2022).

Environmental samples were collected from a 100 cm^2^ area using an InSite^TM^ Rapid Environmental *Listeria species* Test swab (Hygenia, Camarillo, CA, USA) as directed by the manufacturer. Presumptively positive samples were later confirmed using the previously mentioned methods (www.fda.gov/Food/FoodScienceResearch/LaboratoryMethods/ucm071400.htm, accessed on 1 January 2022).

Culture-confirmed isolates were transferred to tryptic soy broth containing 15% glycerol and stored at −80 °C until required.

### 2.2. DNA Isolation

DNA extraction was performed by using the DNeasy Blood and Tissue Extraction Kit (QIAGEN GmbH, Hilden, Germany), from 5 mL of liquid cultures grown overnight at 35 °C in brain heart infusion medium under aerobic conditions, following the manufacturer’s protocol for Gram-positive bacteria. DNA quantity and purity were assessed by using a Nanodrop 2000 spectrophotometer (Thermo Fisher Scientific, Wilmington, DE, USA).

### 2.3. Molecular Confirmation

Isolates were confirmed as *L. monocytogenes* at the Centers for Disease Control and Prevention (CDC) lab (Atlanta, GA, USA) using the AccuProbe *Listeria monocytogenes* Culture Identification Test (Gen-Probe, San Diego, CA, USA).

### 2.4. Serotyping

Serotyping was performed using a commercially prepared antisera according to the manufacturer’s instructions (Denka Seiken, Tokyo, Japan).

### 2.5. PFGE

PFGE analysis was performed according to PulseNet CDC protocol [44]. An unweighted pair-group method with arithmetic mean (UPGMA) dendrogram was generated using BioNumerics ver. 6.6 software (Applied Maths, Sint-Martens-Latem, Belgium) with a Dice coefficient and tolerance of 1.5%.

### 2.6. In Silico Analyses

Library preparation was done using the Nextera XT DNA Sample Kit (Illumina, San Diego, CA, USA). WGS was performed on a MiSeq platform (Illumina) by using 2 × 150 bp runs. After quality control of raw reads, sequences with ≥20× coverage were uploaded in the *Listeria* whole genome multilocus sequence typing database (wgMLST) in BioNumerics version 7.5, (Applied Maths, Sint-Marten-Latem, Belgium), where their sequence type (ST) and lineage were established using wgMLST. Clonal complex (CC) was then assigned using information from the MLST database maintained by the Institut Pasteur, Paris, France. All genomes were deposited in NCBI’s database (see Table S1 for accession numbers). Bioinformatics screens for stress and virulence-associated genes were conducted using ncbi-blast+ version 2.2.30 (National Center for Biotechnology Information, Bethesda, MD, USA). Plasmid and genetic mediators of resistance against sanitizers and heavy metals were identified in genome assemblies using a megaBLAST search with 90% identity and 60% coverage cutoffs. A custom R script was used to parse the best matching BLAST hit with the lowest length score and the highest percent identity. Genomic islands (*LIPI-3*, *SSI-1*) and *comK* were screened using a blastn query with a 90% identity cutoff. *inlA* genomic sequences were identified with a blastn query against all genomes using Geneious software v9 [45]. Multiple sequence alignments were done using the ClustalW plugin.

## 3. Results

### 3.1. L. monocytogenes Identification and Distribution

Of the 63 isolates that were confirmed to be *Listeria*, 58.7% (n = 37) were *L. monocytogenes*, and the remaining 41.3% (n = 26) were identified only to *Listeria* species. Non-*L. monocytogenes* isolates were then excluded from the remainder of the study. A total of 6 *L. monocytogenes* isolates were recovered from facility A (2 environmental and 4 from meat) and 31 from facility B (7 environmental and 24 from meat) (Table 1).

### 3.2. Serotyping

**Figure 1:**
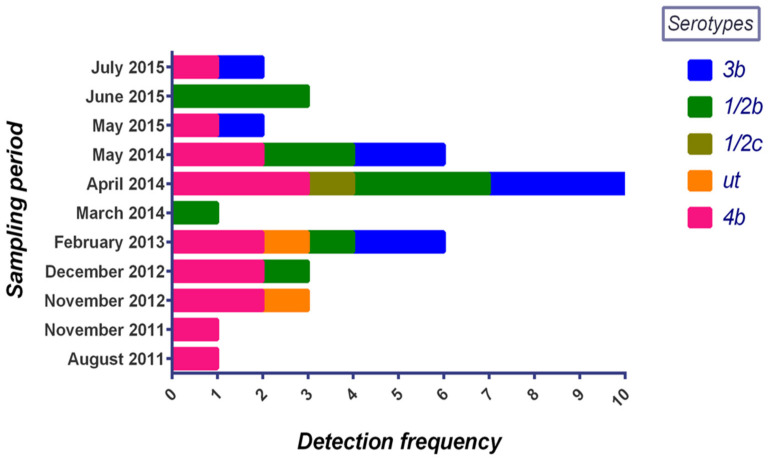
Frequency of *L. monocytogenes* serotypes found in this study between 2011 and 2015. “ut” indicates isolates that are untypeable using the slide agglutination method.

The *L. monocytogenes* isolates were distributed across four serotypes: 1/2c, 1/2b, 3b, and 4b (Figure 1). Serotype 4b showed the highest frequency (n = 14), representing 37.8% of the total isolates. The sources included three raw meat samples and one environmental sample from facility A, along with seven meat and three environmental samples from facility B. Serotype 1/2b isolates comprised the second largest group (n = 11, 29.7%), containing one environmental isolate from each facility, along with nine others that were isolated from meat samples in facility B. Nine (n = 9) isolates (two environmental and seven raw meat-associated) belonging to serotype 3b were recovered from facility B, representing 24.3% of all isolates in this study. Two (n = 2) untypeable isolates were observed in this study, representing 5.4% of the total isolates, and originated from the environment in facility A, and raw meat in B. One (n = 1) serotype 1/2c strain was isolated from raw meat in facility B.

### 3.3. PFGE Analysis

**Figure 2:**
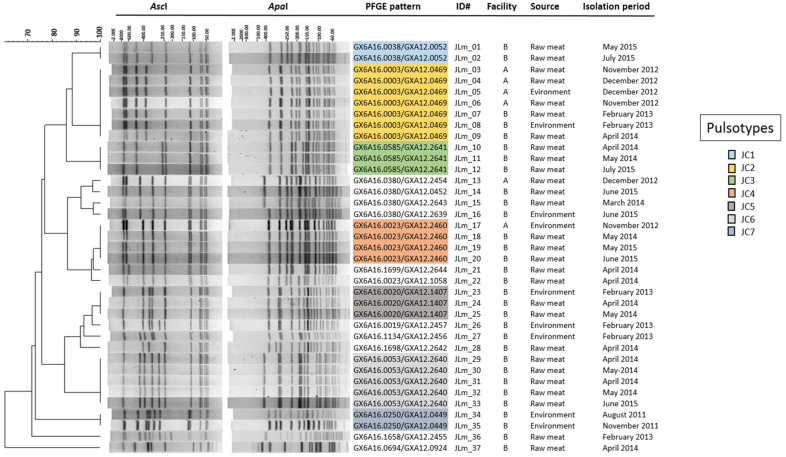
Dendrogram of PFGE pulsotypes from facilities A and B with source and isolation period. Pattern combination clusters are color-coded and indicated by the key.

PFGE typing, using *Asc*I-*Apa*I enzymes, revealed 7 clusters: JC1-7, and a total of 18 pulsotypes across facilities A and B (Figure 2). Isolates JLm_1 and 2 (n = 2) constituted cluster JC1 (PFGE pattern combination GXA16.0038/GXA12.0052) and represented 5.4% of the total isolates. Both isolates originated from raw meat in facilities A and B during May and July 2015, respectively.

Approximately 19% (n = 7) of the isolates, JLm_3-9, were grouped in cluster JC2 (GXA16.0003/GXA12.0469). Three isolates were recovered from raw meat in facility A during November and December 2012, and one environmental isolate in December 2012. In facility B, two isolates belonging to cluster JC2 were recovered from raw meat in February 2013 and April 2014. One environmental isolate which fell into cluster JC2 was also recovered from facility B in April 2014. Cluster JC3 (GXA16.0585/GXA12.2641), composed of isolates JLm_10-12, accounted for 8.1% (n = 3) of all isolates. Cluster JC3 isolates were recovered from raw meat samples in facility B during April–May 2014, and June 2015. Isolates JLm_17-20 represented an overall total of 11% (n = 4) and fell into cluster JC4 (GXA16.0023/GXA12.2460). One isolate was recovered from the environment in facility A during September 2012. The remaining three strains were recovered from raw meat in facility B during May 2014 and 2015, as well as June 2015. Strains JLm_23-25 formed cluster JC5 (GXA16.0020/GXA12.1407), originating from facility B and accounting for ~8% (n = 3) of the isolates. One strain was isolated from the environment in February 2013, and two raw meat-associated strains were recovered during April and May 2014. Five (n = 5) isolates, JLm_29-33, clustered in JC6 (GXA16.0053/GXA12.2640) and originated from raw meat in facility B during April and May 2014, as well as June 2015. Cluster JC6 represented 13.5% of *L*. *monocytogenes* isolates. Cluster JC7 (GXA16.0250/GXA12.0449) comprised 5.4% (n = 2) of the isolates. Both strains, JLm_3 and 35, were recovered from facility B during August and November 2011. The remaining 29.8% (n = 11) of PFGE combination patterns did not form clusters, and accounted for all remaining isolates.

### 3.4. wgMLST Analysis

**Figure 3:**
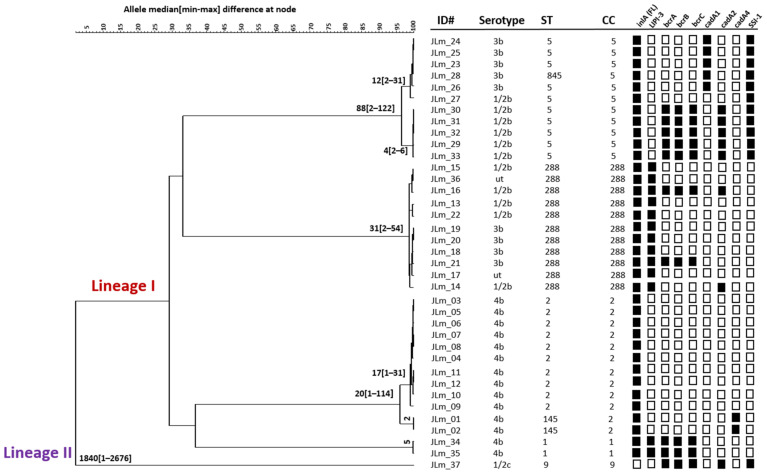
Phylogenetic analysis of 37 *L. monocytogenes* isolates using wgMLST, and distribution of important virulence-associated genes: shaded squares indicate presence of the gene in the corresponding genome; unshaded squares indicate absence.

All isolates in this study clustered into seven sequence types (ST) and five clonal complexes (CC) (Figure 3). Approximately 30% (n = 11) of the isolates fell into ST288, which made up CC288 entirely. wgMLST further discriminated CC288 strains by a maximum of 54 alleles. Serotypes 3b, 1/2b, and two untypeable isolates accounted for the entire CC288 cluster. ST5 accounted for 27% (n = 10) of all isolates, and in addition to a single ST845 isolate, comprised CC5. Two distinct serogroups, 3b and 1/2b, formed CC5. wgMLST further resolved serotype 3b isolates by ≤12 alleles with a median of 12, while 1/2b isolates were differentiated by a maximum of 4 alleles, thus indicating a significant degree of identity. Twelve isolates, 32.4% (n = 12), comprised CC2, which was the largest cluster in this study, constituting ST2 (n = 10) and ST145 (n = 2) strains. All CC2 isolates belonged to serotype 4b and were separated by a maximum of 31 allelic differences by wgMLST. Sequence type 1 (ST1) represented 5.4% (n = 2) of the total isolates, both of which fell into serotype 4b and CC1, and differed by ≤5 alleles, as determined by wgMLST. A single (n = 1) non-lineage I isolate was recovered and fell into ST9 and CC9 according to wgMLST sub-classification. This sole lineage II isolate belonged to serotype 1/2c.

### 3.5. Virulence and Stress-Related Genes

Most isolates from this study (97.3%) encoded the full-length InlA protein (Figure 3), except for isolate JLm_37 (serotype 1/2c), which harbors a deletion at position 12 in the *inlA* gene sequence. The deletion resulted in a truncated predicted InlA protein that is 13 amino acid residues in length (data not shown). Approximately 35% (n = 13) of all isolates possessed the LIPI-3 gene cluster, which encodes for the bacteriocin listeriolysin S (LLS). The ABC transporter cassette *bcrABC* was present in 27% (n = 10) of the isolates. Cadmium resistance genes *cadA1*, *cadA2*, and *cadA4* were also encoded for in 13.5% (n = 5), 21.6% (n = 8), and 5.4% (n = 2) of the isolates, respectively. The gene cluster representing stress survival islet-1 (SSI-1) was found in 32.4% (n = 12) of the isolates.

## 4. Discussion and Conclusions

*L. monocytogenes* isolates that belong to lineage II-associated serotypes are most frequently isolated from food and food production environments globally [17,46,47,48]. However, this study demonstrates a dominant population of isolates that are lineage I-associated serotypes, which colonized and persisted within two Jamaican meat processing facilities during 2011–2015. PFGE analysis identified 18 unique *Ascl*-*Apal* pattern combinations, of which 7 indistinguishable pulsotypes were observed in both facilities across the study period. These subgroups were further resolved into seven ST and five CC groups by classical 7-gene MLST and wgMLST, respectively. Overall, 97% of the isolates in this study possessed *inlA* that encode for the full-length protein. This observation exceeded rates reported from other studies involving food-associated strains of *L. monocytogenes* [16,49], and may indicate increased virulence in these isolates by mediating enterocyte entry through interactions between full-length InlA and the cellular receptor, E-cadherin (Camejo, A. et al., 2011) Furthermore, genetically similar strains were observed within both facilities, suggesting either cross-contamination between facilities, contamination through a common source, or widespread distribution of specific strains in the natural environment.

The relatively high recovery rate of lineage I-associated serotype 4b (37.8%) among the isolates from this study is surprising since these isolates would be expected to be comparatively less prevalent in foods and food processing environments [2,10]. Interestingly, PFGE analysis delineated the 4b isolates into four subgroups (JC1, JC2, JC3, and JC7), inferring additional epidemiologic similarities and differences, despite the isolates originating from both facilities across a ~3.5-year period. However, MLST assigned most 4b isolates to ST2, and all to CC2, thus highlighting a potential epidemiological link that was undetected by PFGE. These findings are in line with previous studies identifying CC2 as a food-associated subgroup within lineage I-associated serotype 4b [50,51]. Interestingly, CC2 was the only subgroup in which no isolates encoded *bcrABC*. Lachtara et al. reported the same observation for CC2 isolates originating from a food processing plant in Poland, and similar to this study, the SSI-1 islet was also absent from these samples [52]. It is noteworthy that the CC2 isolates also belong to the epidemic clone IV subgroup (ECIV, formerly ECIa), which is globally disseminated and has been implicated in multiple outbreaks [13,53,54,55]. MLST analysis further differentiated ST2 strains by ≤31 alleles, underscoring a high degree of genetic similarity within this subgroup, which was also persistent in both facilities over a 3-year period. These observations indicate the persistence of ST2 in both facilities and possible cross-contamination between meat and the environment, potentially arising from movement of the clones between facilities, or their consistent re-entry. The movement of personnel, equipment, and food ingredients is known to play a role in the transmission of *L. monocytogenes* in food-processing facilities [2,56]. It is not known whether there is movement of personnel, equipment, or food-related products between both facilities; however, it is commonplace for skilled workers to undertake projects for multiple processors simultaneously. All CC2 strains found in this study also encoded for the full-length InlA protein.

Isolates belonging to CC288 were distributed across both facilities and were consistently recovered from meat and the environment across multiple years. Only 4 of 11 CC288 isolates comprised pulsotype JC4, underscoring the limitation of PFGE to adequately resolve epidemiologically-related isolates. In contrast, wgMLST analysis showed that all CC288 isolates differed by ≤54 alleles and were closely related. Taken together, these data suggest that CC288 isolates were introduced into both facilities, either through a common source or via cross-contamination, and appear to have persisted within facility B for over a year. Furthermore, all CC288 isolates harbored the LIPI-3 gene cluster, and two also possessed the *bcrABC* resistance cassette. *bcrABC* and LIPI-3 are associated with increased environmental survivability via benzalkonium chloride resistance, and bacteriocin production [26,57,58], and may help to promote the persistence of these isolates in both facilities. Full-length inlA was also encoded in the genomes of all CC288 strains from this study. CC288 isolates are prevalent throughout North America and Europe, and are often associated with food contamination [53,59]. Moreover, CC288 isolates have also been implicated in invasive disease in Asia [60] and should be monitored in food production environments.

Pulsotypes JC5 and JC6, along with three epidemiologically-unrelated isolates according to PFGE, fell into CC5 using wgMLST designation. Interestingly, the CC5 subgroup was very diverse and comprised isolates belonging to serotypes 3b and 1/2b that originated from raw meat and the environment across ~3 years. Similar to PFGE, wgMLST differentiated CC5 into two subgroups, thus highlighting a limitation of classical 7-gene MLST, which categorized almost all CC5 isolates as ST5. Each CC5 subgroup contained homogenous populations of serotypes 3b and 1/2b. The high level of genetic relatedness among isolates within this 3b cluster (separated by ≤31 alleles) indicates the persistence of the subgroup within facility B across ~1 year. Moreover, these isolates all possess the *cadA1* gene that encodes for cadmium resistance and is associated with persistent *L. monocytogenes* strains in a meat producing facility [30]. Although the sampling of both facilities commenced in 2011, CC5 isolates were never isolated from either facility until 2013, indicating the late introduction of this subgroup into the production facilities. Interestingly, the SSI-1 islet was found almost exclusively in serotype 1/2b and 3b strains in this study, an observation that is consistent with reports of SSI-1 having a higher prevalence in non-serogroup 4 strains [61]. Importantly, CC5 isolates belong to ECVI and have been detected in outbreaks involving cantaloupes and ice cream in the USA [62,63]. Other reports have shown CC5 to be highly diverse and widely disseminated throughout South/Central America and Europe [53,64].

PFGE and MLST both identified two epidemiologically-related environmental isolates in facility A across four months during 2011. Both isolates belong to serotype 4b and CC1, were highly genetically similar (≤5 allelic differences), and likely represent the environmental persistence of the strain. CC1 isolates belong to ECI and can exhibit hyper-virulence in human and animal infections [51,65], despite a recent study reporting a lesser association between human disease and CC1 [50]. Nonetheless, ECI isolates have been implicated in global outbreaks [14,15] and are also widely disseminated, particularly across Europe, Asia, and Oceania [53,66,67,68]. Surprisingly, only one lineage II isolate was found during the study, despite the strong association between this lineage and food [1,16,17]. The lone serotype 1/2c isolate, recovered from meat, was sub-classified as ST9 and CC9 by MLST, and possessed a type-4 PMSC [69] in its *inlA* gene sequence. *L. monocytogenes* strains possessing PMSC in *inlA* are still capable of invading intestinal epithelial cells [70], despite being associated with diminished virulence. CC9 clones are prevalent in food and food processing environments, and have also been implicated in sporadic infections [53,66,67].

The control and surveillance of *L*. *monocytogenes* and other foodborne pathogens is an important activity in the Caribbean region for providing safe foods for local consumption, as well as ensuring the viability of key economic sectors such as tourism and export. Jamaican food safety regulations stipulate strict policies for food production and handling that are in line with international standards [71,72], and compliance with regulations and verification of sanitation programs are important for reducing the colonization and spread of *L. monocytogenes* in meat processing facilities [56,73]. A key finding of this study is the propensity of multiple lineage I subtypes of *L. monocytogenes* to thrive in an environmental niche that is usually dominated by lineage II strains. Similar observations involving dominant 4b strains in food and food processing environments have been reported in two South American countries [74,75,76]. Traditionally, Caribbean countries have strong trade ties with Central and South American states. Suppliers of meat and other raw ingredients used in food processing can act as potential sources of contamination [77,78,79,80]. Indeed, it would be interesting to investigate the potential movement of genetically similar lineage I isolates between trading partners within these geographical locations.

Finally, the implementation of more robust monitoring, surveillance, and risk assessment strategies will help to determine the baseline prevalence and epidemiology of *L. monocytogenes* in food production within the Caribbean and other developing regions. These strategies should focus on identifying likely sources of *L. monocytogenes* contamination and the mechanisms and routes of their dissemination. Improved cleaning and sanitation programs within food processing facilities may also help to prevent or stem any trend of persistence and spread of *L. monocytogenes* between food and the food processing environment.

## Figures and Tables

**Table 1:** Total number of samples and collected and identified from different sources in facilities A and B.

Facility	Source	Total Samples	*L. monocytogenes*	*Listeria* spp.
A	Raw meat	6	4	1
	Environment	2	2	0
B	Raw meat	53	24	23
	Environment	12	7	2

## Data Availability

All other information related to this study is available from the corresponding author following a reasonable request.

## Supplementary Materials

The following supporting information can be downloaded at https://www.mdpi.com/article/10.3390/microorganisms10051070/s1, Table S1: *Listeria monocytogenes* isolate ID and NCBI genome accession numbers.
